# Supplementary material for: Regionally Compatible Individual Tree Growth Model under the Combined Influence of Environment and Competition
Source: Plants (Basel). 2023 Jul 19;12(14):2697. doi: 10.3390/plants12142697 (PMC10385731; doi:10.3390/plants12142697)
Supplement: Supplementary file 1 [file plants-12-02697-s001.zip › plants-2328877-supplementary.pdf]

Supplementary Materials

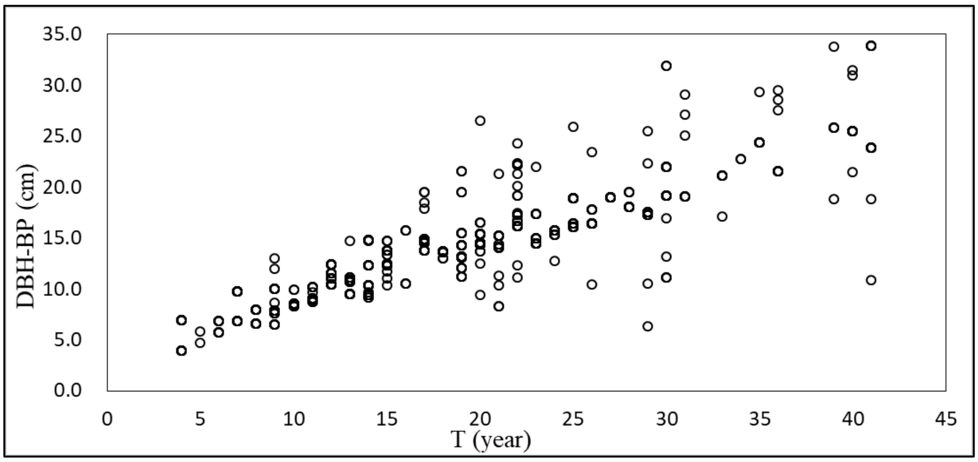

Figure S1. DBH-BP model predicts the change of DBH with T.

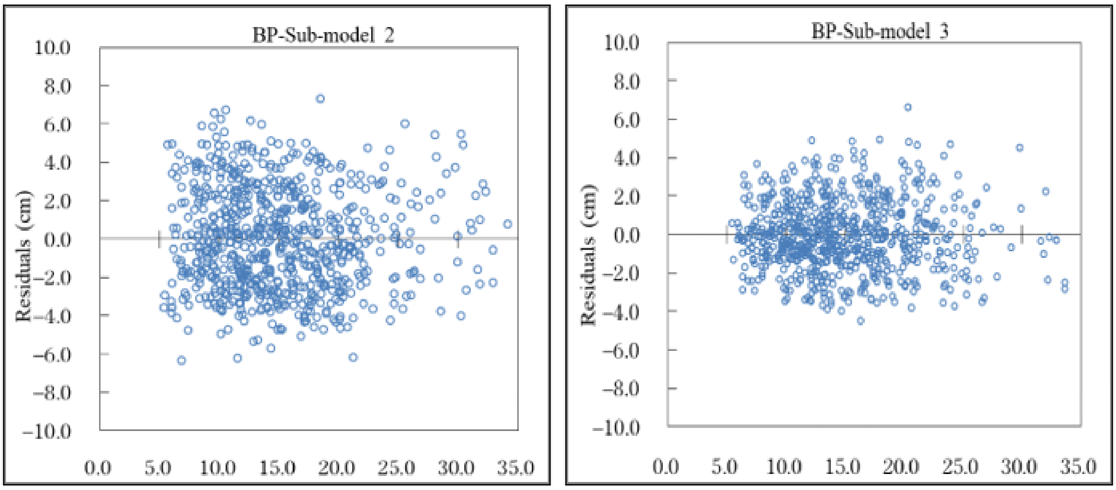

Figure S2. Residual diagram of sub-model 2 and sub-model 3 based on BP model.

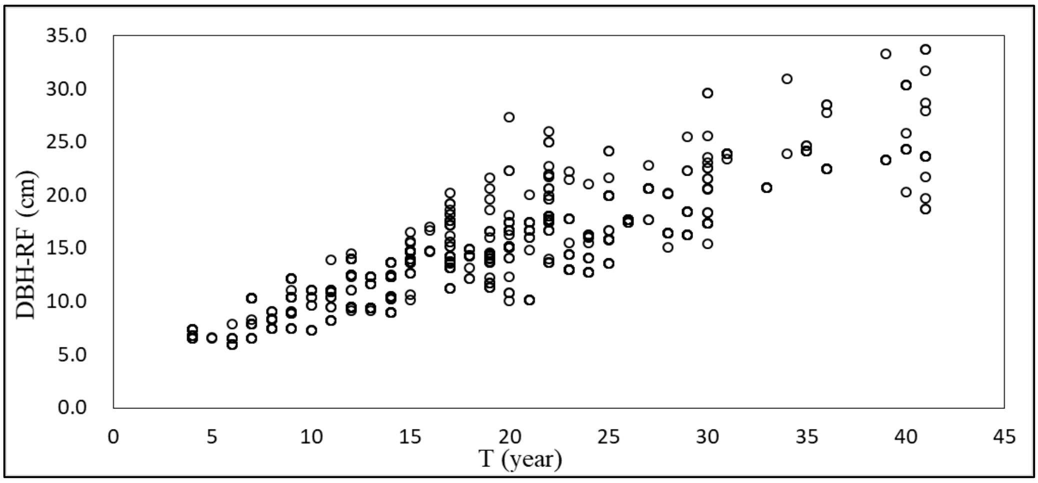

Figure S3. DBH-RF model predicts the change of DBH with T.

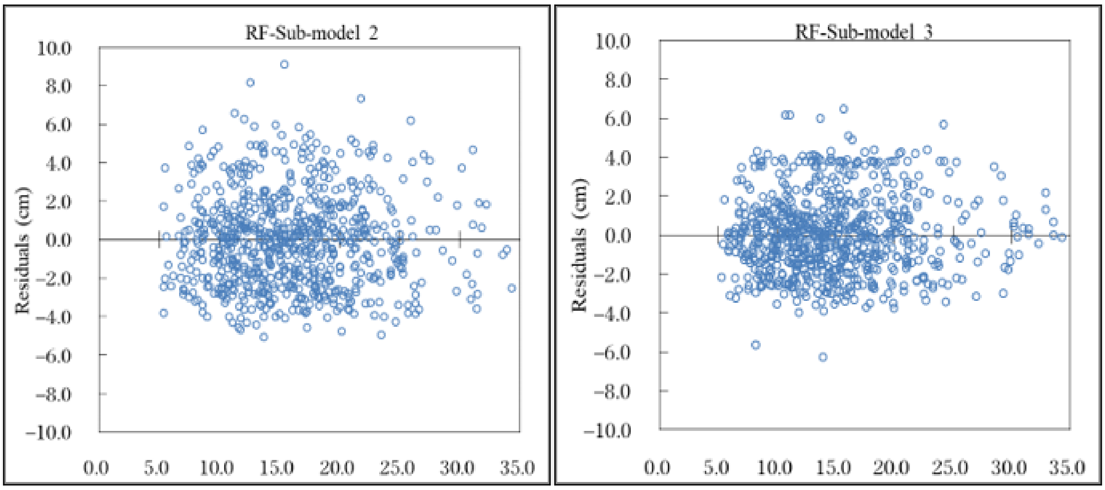

Figure S4. Residual diagram of sub-model 2 and sub-model 3 based on RF model.

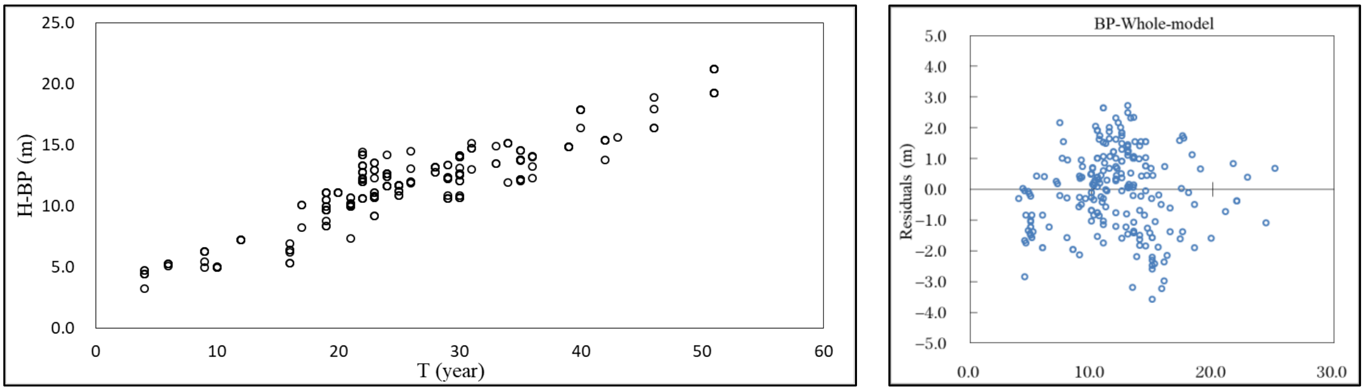

Figure S5. H-BP model predicted H changes with T and residual distribution diagram.

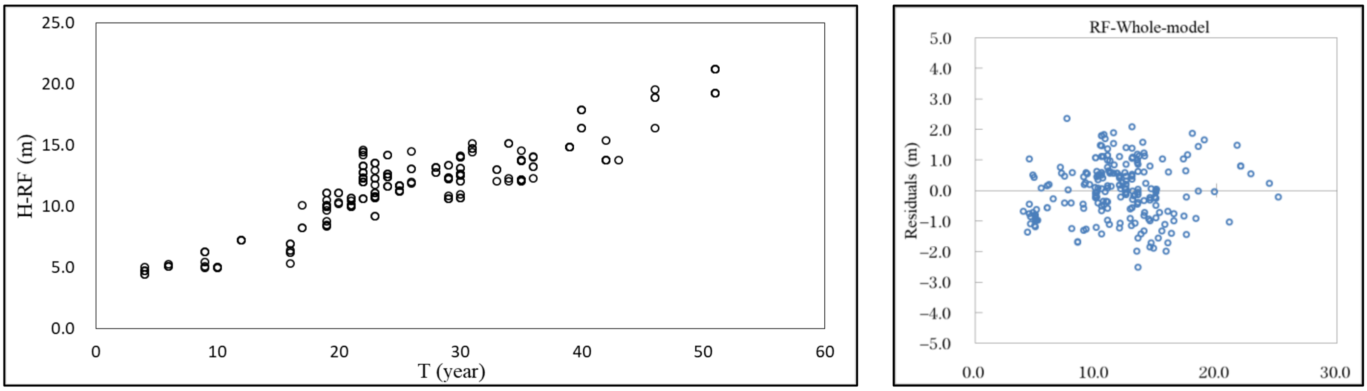

Figure S6. H-RF model predicted H changes with age and residual distribution diagram.

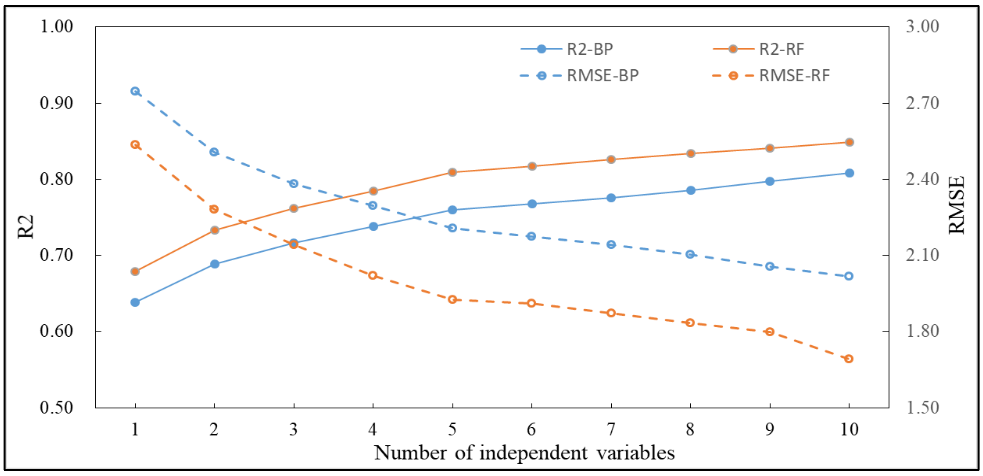

Figure S7. Accuracy changes of BP and RF models with the number of variables.

Table S1. DBH basic model parameters fitting value results and fitting index results.

**Model**	**Parameter fitting value**				**Fitting index**	
	**Parameter**	**a**	**b**	**c**	**R²**	**RMSE(cm)**
Gompertz	Parameter estimate	41.779	1.917	0.030	0.501	3.316
	Lower limit of parameter	30.941	1.743	0.023		
	Upper limit of parameter	52.618	2.099	0.038		
	Standard deviation	5.525	0.090	0.004		
Logistic	Parameter estimate	35.917	4.361	0.054	0.487	3.387
	Lower limit of parameter	30.435	3.806	0.045		
	Upper limit of parameter	41.401	4.920	0.061		
	Standard deviation	2.796	0.285	0.004		
Mitscherlich	Parameter estimate	30.502	0.036	-	0.553	3.102
	Lower limit of parameter	28.559	0.029	-		
	Upper limit of parameter	32.443	0.039	-		
	Standard deviation	0.989	0.002	-		
Richards	Parameter estimate	Un-convergent			-	-
	Lower limit of parameter					
	Upper limit of parameter					
	Standard deviation					

Table S2. H basic model parameters fitting value results and fitting index results.

**Model**	**Parameter fitting value**			**Fitting index**	
	**a**	**b**	**c**	**R²**	**RMSE(m)**
Gompertz	21.905	1.803	0.041	0.647	2.264
Logistic	20.113	3.615	0.062	0.636	2.314
Mitscherlich	20.917	0.036	-	0.656	2.213
Richards	Un-convergent			-	-

Table S3. Precision comparison of the three models in the DBH test date set and H test date set.

Model	D Test Evaluation			H Test Evaluation		
	R^2	RMSE(cm)	MAE(cm)	R^2	RMSE(m)	MAE(m)
RP	0.621	2.813	2.134	0.683	2.102	2.016
BP	0.808	2.017	1.487	0.731	1.857	1.686
RF	0.849	1.691	1.267	0.845	1.267	1.153

Table S4. List of annual climate variables.

Variable	Description	Statistic		
		Maximum	Minimum	Average
**MAT(°C)**	Mean annual temperature	22.2	15.7	19.2
MWMT(°C)	Mean warmest month temperature	30.6	24.6	28.1
MCMT(°C)	Mean coldest month temperature	10.6	5.3	8.5
TD(°C)	temperature difference between MWMT and MCMT	22.1	16.1	19.6
MAP(mm)	Mean annual precipitation	2323	1006	1575
AHM	Annual heat-moisture index	30.1	12.6	19.4
NFFD(day)	Number of frost-free days	360	334	351
Eref(mm)	Hargreaves reference evaporation	1441	1137	1334
CMD(mm)	Climatic moisture deficit	524	35	263
RH(%)	Relative humidity (%)	77	65	70
DD_0(day)	Degree-days below 0°C	6	0	2
DD5(day)	Degree-days above 5°C	6232	3925	5163
DD_18(day)	Degree-days below 18°C	1558	422	952
DD18(day)	Degree-days above 18°C	1969	719	1403
PAS(mm)	Precipitation as snow	6	0	2
EMT(°C)	Extreme minimum temperature over a 30-year period	-0.1	-5.5	-2.1
EXT(°C)	Extreme maximum temperature over a 30-year period	37.5	33.2	36.1

Table S5. Format of DBH data set.

ID	DBH	T	N	DM	HB	PX	PD	PW	TRMC	TRHD	MAT	MWMT	(Etc.)	CMD	RH
1	18.8	24	169	4	607	1	30	2	12	85	19.2	28.8	…	229	72
2	7.6	9	135	5	450	4	22	4	12	35	18.7	27.7	…	225	74
…	…	…	…	…	…	…	…	…	…	…	…	…	…	…	…
1189	11.6	16	146	3	420	4	25	2	13	80	18.9	28.2	…	221	73

[1] (Etc.) represents the remaining 13 climate factors.
